# Anodal frontal tDCS for chronic cluster headache treatment: a proof-of-concept trial targeting the anterior cingulate cortex and searching for nociceptive correlates

**DOI:** 10.1186/s10194-018-0904-9

**Published:** 2018-08-20

**Authors:** Delphine Magis, Kevin D’Ostilio, Marco Lisicki, Chany Lee, Jean Schoenen

**Affiliations:** 1 Headache Research Unit, University Department of Neurology CHR, CHU de Liège, Boulevard du 12ème de Ligne 1, 4000 Liège, Belgium; 2 Department of Biomedical Engineering, Hanyang University, 222 Wangsimni-ro, Seongdong-gu, Seoul, 04763 South Korea

**Keywords:** Chronic cluster headache, Transcranial direct current stimulation, Subgenual anterior cingulate cortex

## Abstract

**Background:**

Percutaneous occipital nerve stimulation (ONS) is effective in refractory chronic cluster headache (rCCH) patients. Responders to ONS differ from non-responders by greater glucose metabolism in subgenual anterior cingulate cortex (sgACC). We reasoned that transcranial direct current stimulation (tDCS), a non-invasive approach, might be able to activate this area and thus improve rCCH patients. Our objective was to explore in a pilot trial the therapeutic potential of tDCS (anode at Fz, cathode over C7) and its possible effects on pain perception, frontal executive functions and mood in rCCH patients.

**Methods:**

Thirty-one patients were asked to apply daily 20-min sessions of 2 mA tDCS for 4 or 8 weeks after a 1-month baseline. CH attacks were monitored with paper diaries. The primary outcome measure was change in weekly attacks between baseline and the last week of tDCS. Twenty-three patients were available for a modified ITT analysis, 21 for per-protocol analysis. We also explored treatment-related changes in thermal pain thresholds and nociceptive blink reflexes (nBR), frontal lobe function and mood scales.

**Results:**

In the per-protocol analysis there was a mean 35% decrease of attack frequency (*p* = 0.0001) with 41% of patients having a ≥ 50% decrease. Attack duration and intensity were also significantly reduced. After 8 weeks (*n* = 10), the 50% responder rate was 45%, but at follow-up 2 weeks after tDCS (*n* = 16) mean attack frequency had returned to baseline levels. The treatment effect was significant in patients with high baseline thermal pain thresholds in the forehead (*n* = 12), but not in those with low thresholds (*n* = 9). The Frontal Assessment Battery score increased after tDCS (*p* = 0.01), while there was no change in depression scores or nBR.

**Conclusion:**

tDCS with a Fz-C7 montage may have a preventive effect in rCCH patients, especially those with low pain sensitivity, suggesting that a sham-controlled trial in cluster headache is worthwhile. Whether the therapeutic effect is due to activation of the sgACC that can in theory be reached by the electrical field, or of other prefrontal cortical areas remains to be determined.

## Background

Cluster headache affects 0.2–0.3% of the general population [1] and is characterized by attacks of excruciating unilateral periorbital/temporal pain associated with ipsilateral autonomic symptoms, lasting 15 to 180 min. 70–80% of patients have the episodic form of the disorder where attacks occur in bouts (clusters) lasting some weeks or months separated by periods of remission of ≥1 month with a circannual periodicity (ICHD-3 beta 3.1.1) [2]. The remaining patients suffer from chronic cluster headache (CCH) where remissions are inexistent or last < 1 month (ICHD-3 beta 3.1.2 [2]). CCH is a dreadful and highly disabling condition, for which available pharmacological treatments [3] often become ineffective and/or induce intolerable side effects. Such refractory patients (rCCH) represent up to 10% of the CCH population [4] and have a high incidence of depression [5], severe sleep disruption, and suicide [6].

Various surgical therapies, including destructive lesions of the trigeminal nerve or the sphenopalatine ganglion, have therefore been applied with disappointing results in terms of efficacy and/or adverse effects [7]. More recently, non-destructive neurostimulation techniques like deep hypothalamic brain stimulation [8], occipital nerve stimulation (ONS) [9] or sphenopalatine ganglion stimulation [10] were found effective in a proportion of rCCH patients. These methods, however, are invasive, may cause serious adverse events [11, 12] and are not universally accessible, partly because of their high cost and need for surgical expertise [11].

Neuroimaging studies clearly suggest that the ipsilateral postero-ventral hypothalamus plays a seminal role during cluster headache attacks [13, 14]. Between attacks, however, there is evidence that frontal brain areas, including the medial frontal [15] and cingulate gyri [16], are dysfunctioning, suggesting a deficient top-down pain control. The precise mode of action of the various neurostimulation techniques in rCCH is not fully understood, but neuroimaging studies provide some insight into possible mechanisms. Using FDG-PET we found that the only difference in brain metabolism between responders and non-responders after 3 and 6 months of ONS treatment was increased glucose uptake in the subgenual portion of the anterior cingulate cortex (sgACC) in responders [16]. Besides other cortical and subcortical structures, the ACC and adjacent inferior medial frontal cortex also showed respectively increased blood flow during hypothalamic deep brain stimulation on H_2_^15^O-PET [17] and connectivity with the effective hypothalamic surgical target on fMRI [18].

Transcranial direct current stimulation (tDCS) is able to directly activate (under the anode) or inhibit (under the cathode) the underlying cerebral cortex. Since its first description by Nitsche & Paulus in 2000 [19], tDCS has been widely studied in a number of neurological and psychiatric disorders [20, 21], including migraine [22, 23], with varying results and an excellent safety profile [24]. The effects of tDCS on the brain might be more complex than initially thought. Most importantly, tDCS can induce changes in brain areas remote from the electrode location. Besides preferential spread of the electric field to the depth of sulci rather than to the surface of cortical gyri [25], tDCS can influence deep structures trans-synaptically including the cingulate cortex [26, 27] and modify cortico-subcortical functional connectivity [27, 28]. Moreover, when applied daily for several days, tDCS is able to modify perceptual functions for several weeks [29, 30]. In an electrophysiological study, tDCS over the primary motor and dorsolateral prefrontal cortex decreased the amplitude of nociceptive laser-evoked potentials [31] and in an FDG-PET study, daily tDCS (20 min, 2 mA) over the motor cortex for 10 days to treat neuropathic pain significantly increased metabolism in the subgenual anterior cingulate cortex [32].

Given the imaging results in ONS responders and the known anatomical spread of tDCS-induced effects, we found it worthwhile to explore in a pilot-trial the therapeutic potential of anodal tDCS over the frontal cortex in rCCH, hypothesizing that it would be able to activate the sgACC, i.e. the area of the brain metabolically activated in clinical responders to ONS therapy [16]. We combined the clinical evaluation with quantitative sensory testing and nociceptive blink reflex recordings searching for possible tDCS-induced changes in pain processing, as well as with an assessment of frontal functions and mood.

## Methods

### Patients

Thirty-one patients (9 females) suffering from rCCH were recruited in our headache clinic (University Department of Neurology, CHR Citadelle, Liège, Belgium). Six patients dropped out during the first week of tDCS treatment because of local skin abrasion and/or inefficacy (*n* = 4), or unrelated health problems (*n* = 2). Two patients did not perform the treatment. These 8 patients were not included in the efficacy analysis.

**Fig. 1:**
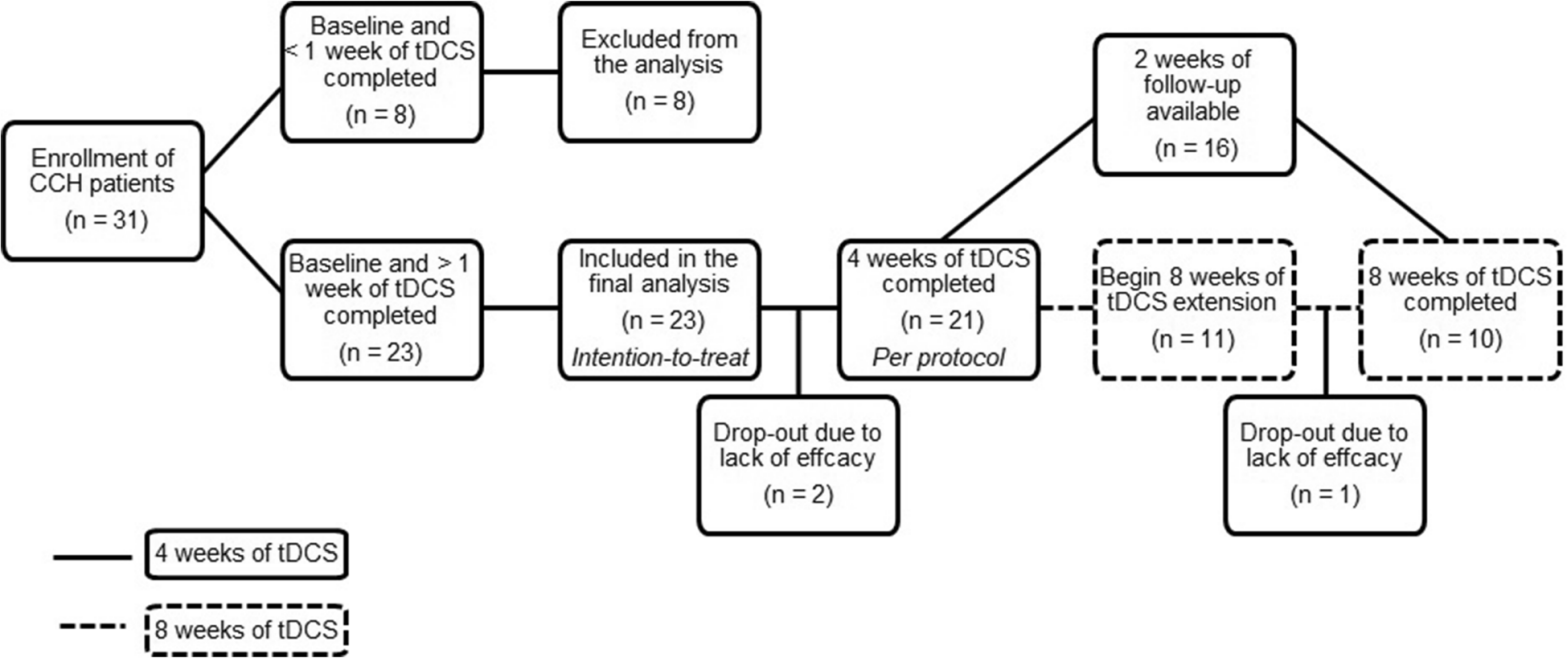
Study flowchart

All patients suffered from the chronic form of cluster headache (CCH, ICHD 3 beta 3.1.2 [2]) (mean chronic phase duration: 11 ± 9 yrs) and had been refractory to at least 3 adequate preventive treatments [4], including methylprednisolone, verapamil, lithium carbonate, topiramate and suboccipital betametasone-lidocaine infiltrations. At the beginning of the study, 19 out of the 23 patients were under preventive treatment (stable for at least 2 months) and were allowed to continue it throughout the trial. One patient had percutaneous occipital nerve stimulation for 8 years (Tables 1 and 2). To be included in the trial, patients had to provide a 4-week headache baseline paper diary and to suffer at least 4 CH attacks per week. Other inclusion criteria were absence of other significant medical or psychiatric conditions and personal or family history of seizures. The 23 patients who treated themselves for more than a week (mean age: 49 ± 10 yrs.; 3 females; mean disease duration: 14 ± 8 yrs) were included in the intention-to-treat (ITT) analysis. Twenty-one out of them achieved 4 weeks of treatment while two patients dropped out before this time period because of treatment inefficacy. The 10 patients first enrolled among the 21 stopped tDCS treatment after 4 weeks and were followed for 2 weeks afterwards. The 11 following patients continued tDCS for another 4 weeks to complete a total treatment of 8 weeks, except for 1 patient who dropped out due to lack of efficacy. In this sub-group, subsequent follow-up information was available in 6 patients. Thus, 21 patients (mean age 49 ± 10 years) were available for a per-protocol (PP) analysis of a 4-week treatment effect, and 10 patients for a PP analysis of 8 weeks of treatment. The patients’ allocation and disposition are depicted in Fig. 1.

**Table 1:** Clinical characteristics of patients included in the analysis

Patients	Age (years)	Gender	CH Side	Baseline weekly attack frequency	CH duration (years)	Chronic phase duration (years)	Ongoing prophylaxis at time of tDCS
1	56	F	R	5	6	2	verapamil - lithium
2	35	M	R/L	30	17	8	verapamil - lithium
3	48	M	R	12	13	13	verapamil - lithium
4	60	M	L	39	9	9	verapamil- clomipramine
5	51	M	L	4	10	10	none
6	46	M	R	7	13	3	carbamazepine - amitriptyline
7	55	M	R	9	20	16	verapamil
8	57	M	L	13	?	?	none
9	56	M	R	13	9	9	duloxetine
10	50	M	R/L	11	18	18	clomipramine
11	41	M	R	12	1.5	1.5	none
12	29	M	R	5	4	4	lithium
13	57	M	L	16	21	18	topiramate
14	50	F	L	4	2.5	2.5	lithium carbonate
15	48	M	L	8	5	3	verapamil - lithium - melatonin
16	59	F	R	60	22	14	ONS
17	63	M	R	17	15	1	verapamil
18	42	M	R	8	16	16	verapamil
19	30	M	R	5	11	5	verapamil - lithium - topiramate
20	53	M	R	18	40	40	verapamil
21	59	M	L	25	14	14	none
22	34	M	L	14	15	15	none
23	40	M	R	22	7	4	verapamil
Mean	48,65				14,25	11,10	
SD	9,97				8,03	8,85	

*CH* cluster headache, *R* right, *L* left, *M* male, *F* female, *ONS* percutaneous occipital nerve stimulation, *tDCS* transcranial direct current stimulation

**Table 2:** Clinical outcome measures: per protocol analysis

	4 weeks tDCS (*n* = 21)		8 weeks of tDCS (*n* = 10)	
	Pre-treatment	Post-treatment	Pre-treatment	Post-treatment
CH attack frequency/week	15,33 ± 13,12	9,91 ± 11,72^***^	18,90 ± 16,01	12,30 ± 16,57^*^
CH attack duration (min)	47.7 ± 50,6	32.6 ± 28.4^*^	32,8 ± 22,0	28,9 ± 28,0
CH attack intensity (0–4)	3.2 ± 0.8	2.5 ± 1.3^*^	2,6 ± 0,7	2,3 ± 1,2
N° of acute treatments/week	13,8 ± 13,8	8,0 ± 8,8^**^	11,9 ± 6,7	5,9 ± 6,2

^*^p < 0.05; ^**^p < 0.01; ^***^p < 0.001

### Transcranial direct current stimulation (tDCS)

**Fig. 2:**
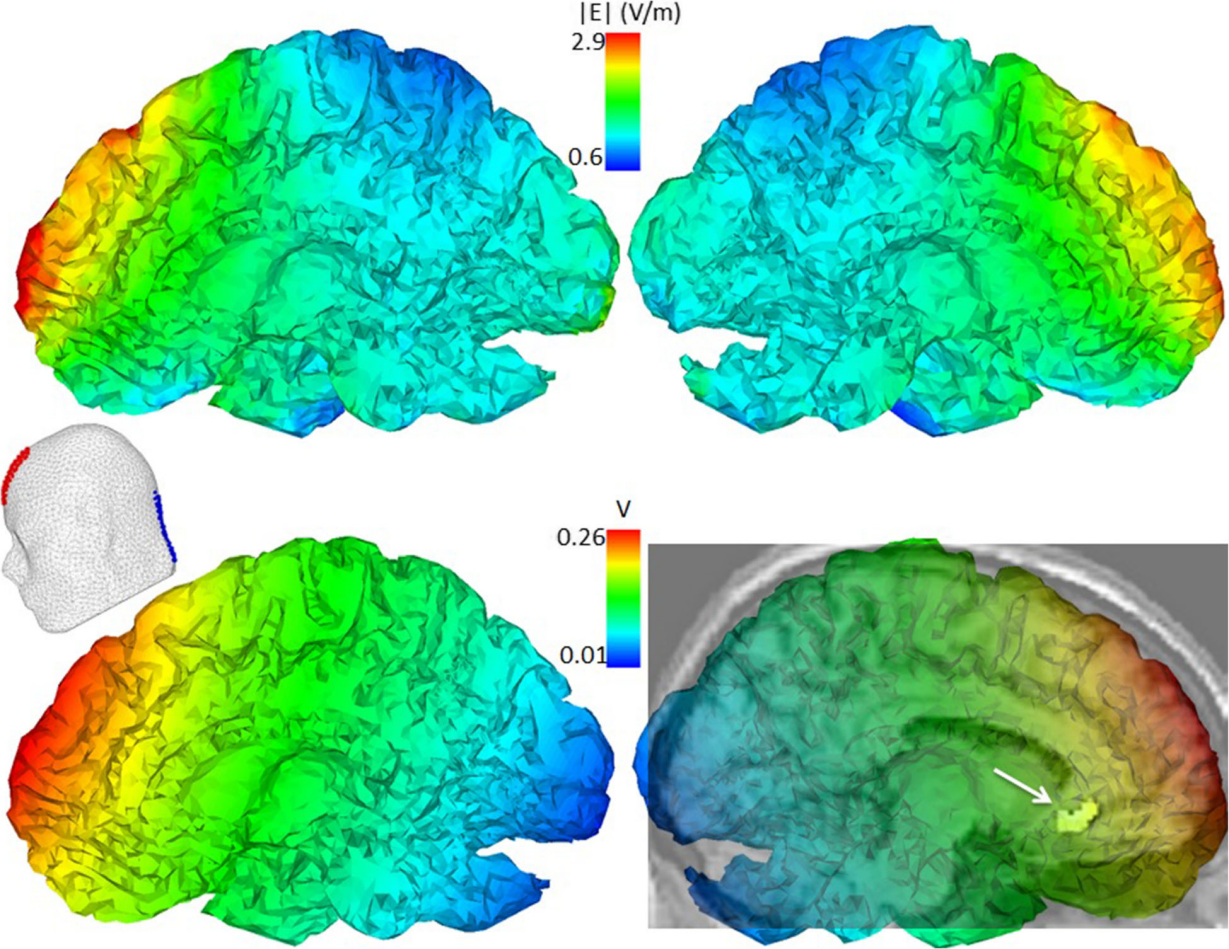
Brain maps of absolute values of electric field intensity (E = V/m) and electric potential (V) in sagittal planes of right and left cerebral hemispheres simulated using COMETS [34] and taking into account tDCS electrode size and placement (insert on the left) as well as current intensity. Lower right: superimposed left sagittal section of a normalized MRI template displaying the subgenual area of the left anterior cingulate cortex (arrow) with increased glucose uptake on FDG-PET in rCCH patients responding to percutaneous ONS compared to non-responders [16]

Bipolar transcutaneous tDCS was applied with a novel portable user-friendly battery-driven device developed by Cefaly Technology® (Seraing, Belgium). The first 4 patients were provided with sticking electrodes containing a special conductive gel (Spes Medica®, Genova, Italy – anode: 35 × 45 mm, cathode: 40 × 90 mm), but developed a transient electro-chemical skin irritation under the cathode after a few days, therefore the treatment was immediately discontinued in these patients. In subsequent patients we employed sponge-electrodes (80 × 60 mm, Spes Medica®, France), moistened with saline, we had used previously in a migraine study with a non-portable tDCS device [23]. The anode was fixed with elastic straps (width 100 mm, Spes Medica®, France) over Fz (10–20 system), the cathode over the spinous process of C7 (Fig. 2). No local skin irritation was seen with sponge electrodes.

All patients were trained to adequately position the electrodes and use the device before starting the trial. Stimulation intensity was set at 2 mA, and patients were asked to apply tDCS outside an attack as a preventive treatment, once daily during 20 min where after the device switched off automatically. The stimulation parameters were set in accordance with safety recommendations [24, 33]. Adherence to the tDCS treatment was monitored with an in-built software designed by Cefaly Technology®. During the trial patients could treat their CH attacks as usual, the majority of them using injectable sumatriptan and oxygen inhalation.

Simulations of absolute value of electric field intensity and electric potential distributions were performed using COMETS [34], a MATLAB (The MathWorks Inc.) toolbox for simulation of local electric fields generated by tDCS, based on the electrostatic finite element method (FEM). Parameters of tDCS (electrode size and placement as well as current intensity) introduced in the model were those applied to patients (described in detail above). Simulation results were imported in Tecplot® (Tecplot Inc., WA, US) for 3D visualization (Fig. 2).

### Clinical assessment

The patients filled in cluster headache paper diaries at least one month before beginning the trial (baseline), during the whole tDCS 4- or 8-week therapy and at least 2 weeks after the end of the treatment. Attack occurrence, intensity (rated 1-mild to 4-worst), duration (minutes) and use of acute treatment (injectable sumatriptan, oxygen inhalation, analgesics) were recorded.

There are unfortunately no clinical biomarkers of ACC activation. Searching for changes in frontal functions associated with tDCS, we determined in all patients a Frontal Assessment Battery (FAB) score before and after treatment [35]. The FAB consists of six subtests exploring conceptualization, mental flexibility, motor programming, sensitivity to interference, inhibitory control and environmental autonomy [35].

Depression scores were also determined before and after tDCS with Beck’s Depression Inventory (BDI) [36].

Patients were interrogated about possible side effects of tDCS at each visit and asked to immediately inform the Headache Research Unit team in case of any adverse event.

### Nociceptive tests

Eighteen out of 21 patients accepted to undergo thermal quantitative sensory testing (QST) and 11 to have nociception-specific blink reflex (nBR) recordings before and after treatment.

During QST, using a thermode (Advanced Thermal Stimulator-Medoc™ USA), we determined sensory and pain thresholds to cold (CST and CPT) or warm stimuli (WST and WPT) bilaterally over the forehead and the volar side of the wrist. The device allows to deliver stimuli between − 10 °C and + 54 °C. The thresholds were determined in steps of 1 °C/second starting at 32 °C. The subjects were instructed to press a button when they perceived the stimulus and when it became painful. The mean of three successive measures was taken as threshold value for each variable.

Nociception-specific blink reflexes (nsBR) were recorded as previously described [37]. Briefly, surface recording electrodes were placed bilaterally over orbicularis oculi muscles, and electrical stimulation was performed supraorbitally with a concentric electrode (central cathode: 1 mm; insert: 8 mm; anode: 23 mm). Monopolar square pulses of 0.2 ms duration were delivered at a pseudo randomized interstimulus interval between 15 and 17 s. We first determined electrical sensory and pain thresholds using ascending and descending steps of 0.2 mA intensity (Digitimer stimulator DS7A). To elicit the nsBR, the final stimulus intensity was set at 1.5 times the individual pain threshold. Sixteen rectified electromyographic responses were recorded and averaged off-line (CED 1401 and 1902 devices, Signal 4.11 Software, Cambridge Electronic Design, Cambridge, UK). The first response of each session was discarded to avoid contamination with startle responses. The remaining 15 sweeps were averaged in three sequential blocks of five responses. The amplitude of the R2 response was calculated for each block and expressed as area under the curve (AUC). Results were normalized using R2 AUC divided by the square of stimulus intensity (AUC/i^2^). Habituation of the nsBR was calculated as the percentage change of the R2 AUC between the 3rd and the 1st block of averages and also expressed as the regression slope of the R2 AUC over the three successive blocks of five responses.

### Data analysis

The primary outcome measure was the change of weekly CH attack frequency during and following tDCS treatment, compared to the mean weekly frequency during the 4-week baseline. Secondary outcome measures were change in attack intensity and duration, and acute medication use.

As mentioned above, 8 patients were not included in the analysis because they applied tDCS for less than 1 week. Two patients stopped treatment before the 4-week term and were considered protocol violators; their data were handled on a “last value carried forward” basis for the ITT analysis. Twenty-three patients were thus available for intention-to-treat (ITT), 21 for per-protocol (PP) analysis of the effects of daily tDCS treatment during 4 weeks. A subgroup of 10 patients was available for assessing the effect of an 8-week treatment.

PP and ITT outcomes were analysed with the Wilcoxon signed-rank test and Friedman’s Anova (Statistica 8.0, StatSoft, France). The Wilcoxon signed-rank test was also used to compare electrophysiological values and psycho-behavioural scores before and after tDCS. A *p* value ≤0.05 was considered significant.

Like in a study on neuropathic pain [38], QST data were first standardized and then entered in a non-hierarchical K-means cluster analysis. This analysis was employed in order to identify subgroups of patients with distinct sensory profiles and their possible correlation with treatment outcome. We searched if the tDCS treatment effect was correlated with pre-treatment pain thresholds using Pearson’s correlation analysis and if tDCS had an effect on pain thresholds with mixed-design ANOVA.

## Results

### Clinical outcome

**Fig. 3:**
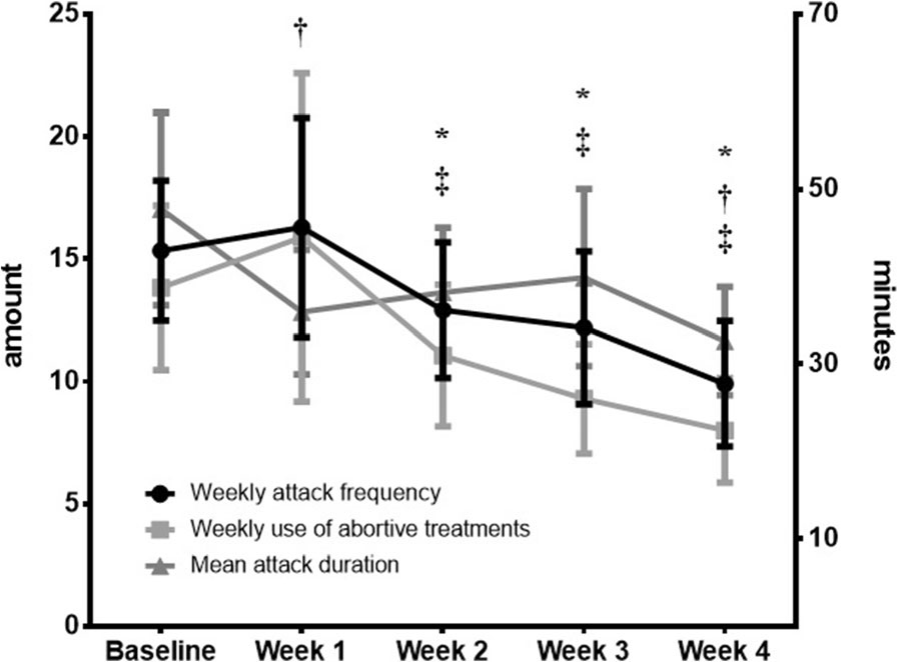
Attack frequency, attack duration and number of attack treatments during 4 weeks of daily tDCS (means ± sem). Significant changes (*p* < 0.05) from baseline are respectively indicated for each item (*), (†), (‡)

The changes in outcome measures in the *per-protocol* (PP) analysis (*N* = 21) over 4 weeks of treatment are graphically depicted in Fig. 3. Mean weekly attack frequency decreased significantly from 15.33 ± 13.12 at baseline to 9.91 ± 11.72 after 4 weeks of tDCS (− 5.43/35%, *p* < 0.001). The 50% responder rate was 38%. Mean attack duration decreased from 47.70 ± 50.55 min at baseline to 32.62 ± 28.38 min (*p* = 0.020) and mean attack intensity from 3.2 to 2.5 (*p* = 0.016). Weekly use of abortive treatments decreased from 13.82 ± 13.83 at baseline to 8.00 ± 8.81 at 4 weeks (*p* = 0.006) (Fig. 3).

**Fig. 4:**
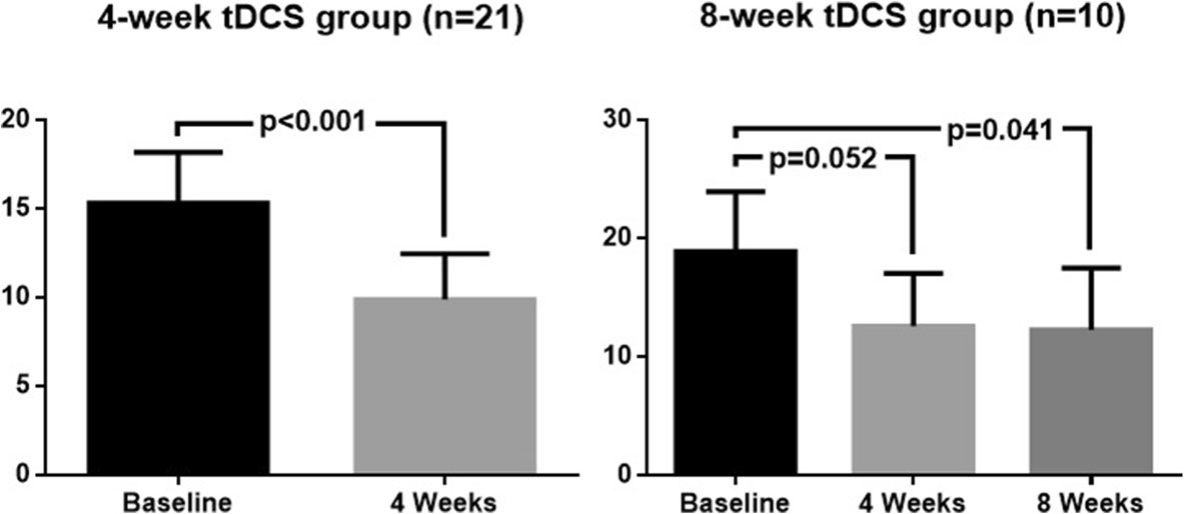
Weekly CH attack frequency at baseline and after 4 weeks (left) and 8 weeks (right) of daily tDCS (means ± sem)

Favourable outcomes were sustained over time (Friedman test *p* = 0.049) (Fig. 3). In the subgroup of patients who treated themselves with tDCS for 8 weeks (*N* = 10), weekly CH attack frequency decreased from 18.90 ± 16.01 at baseline to 12.30 ± 16.57 (*p* = 0.041, Fig. 4). The 50% responder rate was 50%. Reductions in mean attack duration, severity and acute treatment use did not reach the statistical level of significance in this smaller subset of patients.

In the *intention-to-treat* analysis of all patients who performed at least 1 week of tDCS (*N* = 23) the results were similar showing a significant decrease of attack frequency after 4 weeks of treatment (*p* < 0.001).

Follow-up headache diaries were available for 16 patients, as five subjects stopped filling them in after the end of tDCS therapy. In this subgroup of 16 patients, weekly CH attack frequency returned to pre-treatment levels 2 weeks after tDCS (13.38 ± 15.88) despite a significant decrease with respect to baseline during the treatment period (from 15.06 ± 14.59 to 10.81 ± 14.27; *p* = 0.007).

A pooled analysis of compliance revealed that patients who completed the protocol (4 or 8 weeks) had used the tDCS device 87% of the recommended time.

### Nociceptive tests

Overall, thermal QST results were not modified by tDCS whatever modality (cold/warmth), threshold (sensory/painful), side (right/left) or stimulus location (forehead/wrist) was considered (all *p* > 0.1). Along the same line, electrical thresholds and nsBR results were not modified by tDCS (all *p* > 0.1).

**Fig. 5:**
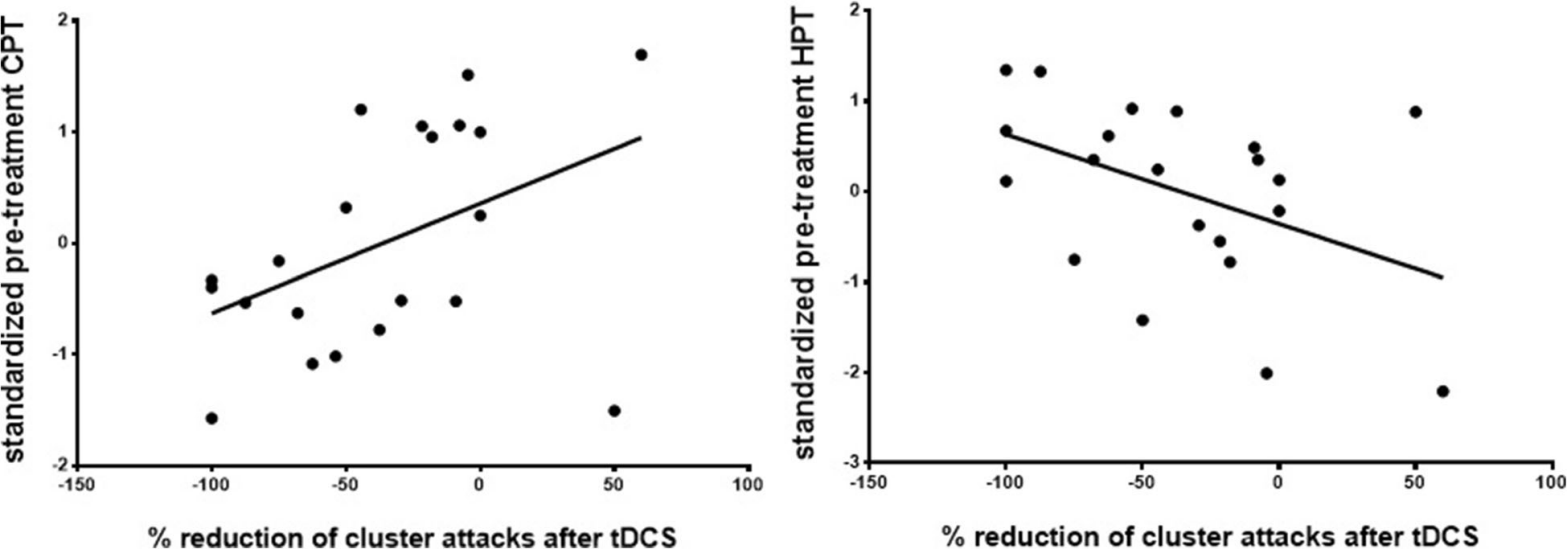
Correlations between the percentage change in weekly CH attack frequency after daily tDCS (baseline vs. week 4) and the baseline standardized cold (CPT) and heat pain thresholds (HPT)

Searching for correlations between treatment response and baseline thermal pain thresholds, we found that patients who perceived pain at more extreme temperatures exhibited a better response to tDCS. Individual baseline cold pain threshold (CPT) correlated with the percentage reduction of attack frequency after 4 weeks of tDCS (*N* = 21, *r* = 0.45, *p* = 0.042) and concordantly, heat pain threshold (HPT) anti-correlated with tDCS-induced attack frequency reduction (*r* = − 0.45, *p* = 0.041, Fig 5). A data-driven K-means cluster analysis revealed 2 distinct QST profiles: patients with low (‘hypersensitive’, *N* = 9) and patients with high pain thresholds (‘hyposensitive’, *N* = 12). The tDCS-induced reduction in CH attack frequency was greater in ‘hyposensitive’ (− 6.67 attacks/week, *p* = 0.014) than in ‘hypersensitive’ patients (− 3.78 attacks/week, *p* = 0.049).

The mean frontal assessment battery (FAB) score significantly increased after tDCS, (from 16.58 ± 1.46 to 17.16 ± 1.17, *N* = 19, *p* = 0.01). There were no significant changes in BDI scores (13.18 ± 18.88 before vs 12.41 ± 9.22 after tDCS, *p* > 0.1).

### Adverse events

The sponge electrodes were well tolerated and did not produce any skin abrasion, like in our previous tDCS study in migraine [23]. Besides a slight and transient tingling sensation at the electrode site, frequently reported with tDCS [24], there were no treatment-related adverse effects. Among the 8 patients who stopped tDCS during the 1st week, 2 had actually not switched on the device at all while the 4 others applied tDCS only for a few days because of electrochemical skin irritation related to the use of sticking electrodes. These electrodes had been tested with the tDCS device by the manufacturer before the study. We hypothesize that the repetition of tDCS could be responsible for this skin irritation. Conversely, sponge electrodes were very well tolerated at long–term. Two patients dropped out for unrelated health problems: ENT cancer and peritonitis.

## Discussion

Our study suggests for the first time that excitatory tDCS over the frontal cortex targeting the anterior cingulate cortex could be a useful non-invasive, well tolerated add-on therapy for attack prevention in patients suffering from chronic cluster headache refractory to preventive treatments (rCCH). After 4 weeks of one daily 20-min session of tDCS there was on average a 37% reduction in weekly attack frequency (− 5.39) and a 50% frequency responder rate of 43%, when patients who completed at least 1 week of treatment were included in the analysis. As this was an open label proof-of-concept trial, we excluded from the outcome analysis subjects who dropped during the first week. Despite the relatively small size of the subgroup of patients who completed 8 weeks of treatment, our study was able to detect a sustained beneficial effect of tDCS on the number of weekly attacks (− 6,60 attacks or 35.6% reduction). As illustrated in Fig. 3, there was overall no significant clinical change during the 1st week of treatment, or even a slight numerical increase in attack frequency. This may suggest that it takes some time for tDCS to induce plastic changes [39] in frontal networks [14, 15]. The lack of improvement during 1st week may also explain some of the early drop-outs and should be explained to patients in future tDCS trials. Future study protocols should also consider extending the treatment period beyond eight weeks, since in the present study clinical improvement did not last for more than 2 weeks after interrupting tDCS.

These outcomes may appear modest at first sight. One has to take into account, however, that rCCH patients are most difficult patients to treat and that tDCS is an accessible and safe therapy devoid of serious adverse effects [40]. There was great variation of attack frequency between patients reflecting clinical practice and of treatment effects, which could in part be related to the known inter-individual variability of physiological tDCS changes [41]. Needless to say that a randomized, sham-controlled trial is warranted to confirm the results of this open label study. Given the excellent safety and tolerability of tDCS, however, such a trial could target a less- or non-refractory population of chronic and episodic cluster headache patients, which might increase the effect size.

Up to now transcutaneous cervical vagus nerve stimulation (nVNS) is the only other non-invasive neurostimulation method that was studied in cluster headache, though not in patients refractory to preventive drugs. Similar benefits were reported with nVNS in CCH attack prevention after 4 weeks of daily stimulations (− 5.9 attacks/week [42]) and for the acute treatment of episodic, but not chronic CH [43].

Baseline scores on the Frontal Assessment Battery (FAB) were non significantly lower (16.58 ± 1.46) in our patients than available normative values matched for age (17.1 ± 1) [44]. After tDCS therapy FAB scores increased significantly. Although we cannot rule out a learning effect, this may be due to an excitatory effect of anodal tDCS on frontal and prefrontal areas that are known to be dysfunctioning in CH according to behavioural [45] and fMRI studies [46].

The fact that tDCS had no effect on thermal pain thresholds or on amplitude of the nociceptive blink reflex suggests that it has no direct anti-nociceptive effect. The therapeutic effect of tDCS, however, was greater in patients with high baseline pain thresholds than in those belonging to the low threshold subgroup. Whether this is related to allodynia that is prevalent during CH attacks and may outlast the attack [47] and/or to different underlying brain activation states known to influence tDCS effects [48] remains to be determined. It suggests nevertheless that baseline pain thresholds could have predictive value for tDCS treatment success in future clinical trials.

The rationale of this proof-of-concept study was that Fz anode-C7 cathode tDCS would be able to activate the anterior cingulate cortex (ACC) of which we found the subgenual portion (sgACC) to be hypermetabolic in rCCH patients responding to percutaneous ONS [16]. Simulations using the COMETS [34] toolbox indicate indeed that our tDCS protocol generates an electric field able to reach this area of the deep frontal cortex. As illustrated in Fig. 2, however, the electrical field generated by tDCS spreads largely over several prefrontal areas that are implicated in cluster headache pathophysiology [14, 15, 18, 45, 46], and may even exert a lesser effect in other subcortical structures like the hypothalamus, known to be pivotal in this disorder [49]. Current density maps suggest that tDCS-related brainstem activation is probably negligible. Moreover, we didn’t observe any signs specific to brainstem modulation (like visual disturbances or vertigo, or nsBR modifications). Although the prefrontal cortex is involved in pain control, a comprehensive review shows that tDCS trials targeting areas such as the dorsolateral prefrontal cortex are overall ineffective in chronic pain disorders [21]. Thus, although increased cortical excitability has been demonstrated in episodic (not chronic) cluster headache patients [50], it is likely that the activation of prefrontal cortices in our tDCS protocol was not involved directly in the beneficial therapeutic effect, but rather via its connexions with subcortical structures including the ACC [25–27]. Unfortunately, we had no access to functional neuroimaging nor laser evoked potentials, which would have allowed a more straightforward anatomo-clinical interpretation.

## Conclusions

To conclude, this proof-of-concept study suggests that daily tDCS (2 mA, 20 min) with the anode at Fz and the cathode at C7 could be a useful and well-tolerated therapy in difficult-to-treat chronic cluster headache patients, refractory to medical treatment. The beneficial effect takes 1 week to appear and is short-lasting after the treatment period. The mechanism of action could be an activation of the subgenual anterior cingulate cortex either directly via the generated electrical field or via activation of prefrontal areas. It remains to be determined if the effect size could be greater in less disabled cluster headache patients. We are aware that these results need to be confirmed in a randomized sham-controlled trial, for which our study has provided several methodological hints.

## Abbreviations

CH: Cluster Headache
CPT: Cold pain threshold
CST: Cold sensory threshold
FAB: Frontal Assessment Battery
ITT: Intention-to-treat
nBR: nociceptive blink reflexes
ONS: Occipital nerve stimulation
PET: Positron emission tomography
PP: Per-protocol
QST: Thermal quantitative sensory testing
rCCH: refractory chronic cluster headache
sgACC: subgenual anterior cingulate cortex
tDCS: transcranial direct current stimulation
WST: Warm sensory threshold
